# Hygiene, Sanitation, and Water: Forgotten Foundations of Health

**DOI:** 10.1371/journal.pmed.1000367

**Published:** 2010-11-09

**Authors:** Jamie Bartram, Sandy Cairncross

**Affiliations:** 1 Water Institute, Gillings School of Global Public Health, University of North Carolina at Chapel Hill, North Carolina, United States of America; 2 London School of Hygiene & Tropical Medicine, London, United Kingom

## Abstract

As the first article in a four-part *PLoS Medicine* series on water and sanitation, Jamie Bartram and Sandy Cairncross argue that the massive burden of ill health associated with poor hygiene, sanitation, and water supply demands more attention from health professionals and policymakers.

**Summary Points**

- A massive disease burden is associated with deficient hygiene, sanitation, and water supply and is largely preventable with proven, cost-effective interventions.
- The total benefits of these interventions are greater than the health benefits alone and can be valued at more than the costs of the interventions.
- Hygiene, sanitation, and water supply are development priorities, yet the ambition of international policy on drinking water and sanitation is inadequate.
- Hygiene, sanitation, and water supply continue to have health implications in the developed world.
- The active involvement of health professionals in hygiene, sanitation, and water supply is crucial to accelerating and consolidating progress for health.

**This is the introductory article in a four-part *PLoS Medicine* series on water and sanitation.**

## 

Globally, around 2.4 million deaths (4.2% of all deaths) [1] could be prevented annually if everyone practised appropriate hygiene and had good, reliable sanitation and drinking water. These deaths are mostly of children in developing countries from diarrhoea and subsequent malnutrition, and from other diseases attributable to malnutrition.

How is an opportunity to prevent so many deaths (and 6.6% of the global burden of disease in terms of disability-adjusted life years or DALYs [1]) failing to attract the attention of the international public health community?

In this introductory paper to the *PLoS Medicine* series on water and sanitation, we develop the idea that these basic needs are the forgotten foundations of health.

## A Massive Disease Burden Is Associated with Deficient Hygiene, Sanitation, and Water Supply

While rarely discussed alongside the “big three” attention-seekers of the international public health community—HIV/AIDS, tuberculosis, and malaria—one disease alone kills more young children each year than all three combined. It is diarrhoea [2], and the key to its control is hygiene, sanitation, and water (HSW).

**Figure 1:**
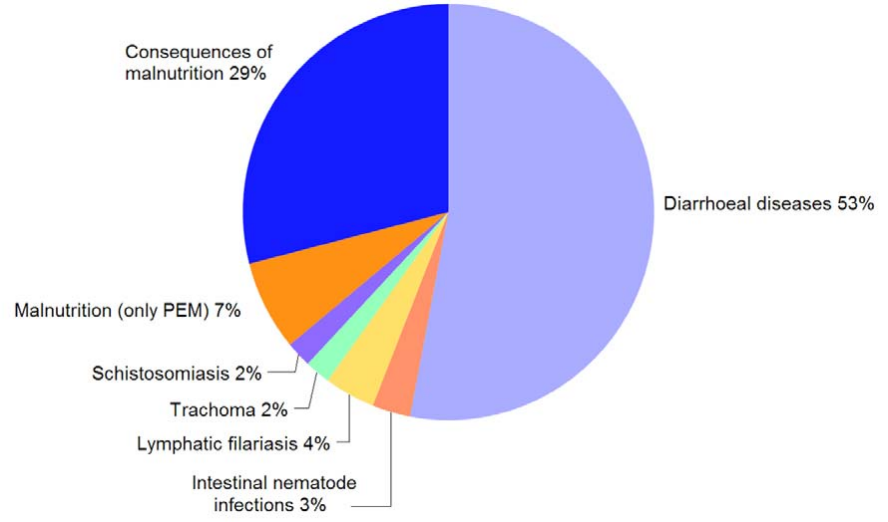
Contributions in DALYs of individual diseases to the total burden of ill-health preventable by improvements in HSW. PEM, protein-energy malnutrition. Source: [1].

Figure 1 breaks down the preventable HSW-associated disease burden. It is dominated by mortality from infectious diarrhoea, nearly 90% of which is borne by children under five years old and 73% of which occurs in only 15 developing countries [1]. Moreover, mortality from diarrhoea is only part of the disease burden. Even using the most conservative scenarios, the long-term sequelae due to diarrhoea in early childhood contribute more DALYs than do the deaths [3].

Regrettably, it is no surprise that much ill health is attributable to a lack of HSW. Globally, nearly one in five people (1.1 billion individuals) habitually defecates in the open. Conversely, 61% of the world's population (4.1 billion people) has some form of improved sanitation at home—a basic hygienic latrine or a flush toilet. Between these two extremes, many households rely on dirty, unsafe latrines or shared toilet facilities [4]. Not only can it prevent endemic diarrhoea, adequate sanitation can help to prevent intestinal helminthiases, giardiasis, schistosomiasis, trachoma, and numerous other globally important infections (Table 1).

**Table 1:** Environmental classification of water- and excreta-related infections.

Category	Examples	Control Strategies
**A. Feco-oral (Potentially water-borne or water-washed)**	*Viral*Hepatitis A, E, and FPoliomyelitisViral diarrhoeas*Bacterial*CampylobacteriosisCholeraPathogenic *E. coli*SalmonellosisTyphoid, paratyphoid*Protozoal*AmoebiasisCryptosporidiosisGiardiasis	Improve water quality (to prevent water-borne transmission), improve water availability, hygiene promotion (to prevent water-washed transmission)
**B. Purely water-washed**	*Skin and eye infections*ScabiesConjunctivitisTrachoma*Louse-borne infections*Relapsing fever	Improve water availability, hygiene promotion
**C. Soil helminths**	AscariasisTrichuriasisHookworm infection	Sanitation, hygiene promotion, treatment of excreta before re-use
**D. Tapeworms**	*Taenia solium* infection*Taenia saginata* infection	As C above, plus meat inspection and cooking
**E. Water-based diseases**	*Bacterial*CholeraLegionellosisLeptospirosis*Helminthic*SchistosomiasisClonorchiasisDracunculiasis	Reduce contact with/consumption of infected water, sanitation, treatment of excreta before re-use
**F. Insect vector diseases**	*Water-related*DengueYellow feverMalariaWest African trypanosomiasis*Excreta-related*Bancroftian filariasisTrachomaFly- and cockroach-borne excreted infections^a^	Reduce number of potential breeding sites and need to pass near them, improve surface water drainage, use repellent/insecticide where appropriate
**G. Rodent-borne diseases**	Rodent-borne excreted infectionsLeptospirosisTularaemia	Rodent control, hygiene promotion, reduce contact with infected water

**Source:** Adapted from [58].

a Excreted infections comprise all those in Categories A, C, and D plus helminthic diseases in Category E.

The situation for drinking water appears better than that for sanitation. Although around 13% of the world's population (884 million people) lives in households where water is collected from distant, unprotected sources, 54% (3.6 billion) receives piped water at home. However, many piped water systems in developing and middle income countries work for only a few hours per day and/or are unsafe. In larger Asian cities, for example, more than one in five water supplies fails to meet national water quality standards [5]. Reliable safe water at home prevents not only diarrhoea but guinea worm, waterborne arsenicosis, and waterborne outbreaks of diseases such as typhoid, cholera, and cryptosporidiosis.

Much of the impact of water supply on health is mediated through increased use of water in hygiene. For example, hand washing with soap reduces the risk of endemic diarrhoea, and of respiratory and skin infections, while face washing prevents trachoma and other eye infections. A recent systematic review of the literature [6] confirmed that hygiene, particularly hand washing at delivery and postpartum, also helps to reduce neonatal mortality. It might be argued that water supplies also make flush toilets feasible, but this does not necessarily add to their health benefits, as we have seen no credible evidence that the health benefits of sanitation cannot be achieved by dry latrines, if they are properly built and maintained [7].

## This Disease Burden Is Largely Preventable with Proven, Cost-Effective Interventions

**Figure 2:**
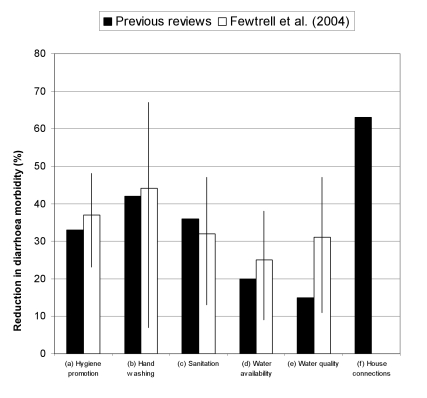
Results of reviews of the effect on diarrhoea of HSW interventions. Results of the previous reviews are for the better quality studies. The reduction for household drinking water connections is in addition to reductions for water quality and availability of public sources. Previous reviews: (a), (c)–(f) [8]; (b) [11]). Fewtrell et al. [57].

Figure 2 shows the average reductions in diarrhoea incidence found to be associated with HSW interventions in several literature reviews. The impact of “real world” interventions varies widely in response to local factors such as which pathogens are contributing to disease and the relative contribution of different transmission routes.

A balanced interpretation of the available evidence suggests that a reasonably well-implemented intervention in one or more of hygiene, sanitation, water supply or water quality, where preexisting conditions are poor, is likely to reduce diarrhoeal disease prevalence by up to a third. Still greater reductions (up to 63%) are associated with water piped to one or more taps on a property [8]. Such a major impact merits far more attention from health professionals and health systems than has been common in recent decades.

We are still learning about the role of HSW in disease control. For example, *Ascaris* and other intestinal worms are known to be associated with poor sanitation, but a recent review [9] found evidence that hand washing with soap can also help to prevent transmission of ascariasis. We know that trachoma is prevented by facial hygiene and hand washing, but recent research has also highlighted the role of latrines in controlling the *Musca sorbens* flies that carry the *Chlamydia* pathogen between children's faces [10]. Even regarding the effect of hygiene on diarrhoea among young children in poor communities, we still have much to learn. There is good evidence to justify promotion of hand washing with soap [11], but for other aspects of hygiene behaviour, such as proper disposal of children's stools [12], the epidemiological evidence is from observational studies, which are subject to confounding.

The most effective means of promoting behaviour change is also a fruitful research field. It has only recently become clear to health professionals that emotional levers (“Clean hands feel good”) change people's health behaviours more effectively than cognitive statements (“Dirty hands cause disease”). Advertising agencies have known this for years. They also know the importance of investing in formative research, testing, and evaluation, to tailor the messages to local people's beliefs and aspirations [13]. If health workers can divest themselves of the unsubstantiated belief that health considerations motivate behaviour, they can become a more effective force for hygiene behaviour change.

There are alternative ways to tackle some of the HSW-associated disease burden. The widespread introduction of oral rehydration therapy (ORT) in the 1980s, for example, contributed much to reducing mortality from diarrhoeal disease [14]. However, such interventions focus on mortality rather than morbidity and on secondary rather than primary prevention. Moreover, ORT does not address the problems of persistent diarrhoea and dysentery.

It is sometimes claimed that the lack of an overall decline in diarrhoea morbidity rates despite increasing coverage with water and sanitation shows that the health benefits of HSW are illusory. However, there are other possible explanations for the apparent contradiction. First, coverage has not advanced as rapidly as one would wish, or as some official figures suggest. Second, the diarrhoea morbidity data are subject to a variety of interpretations; for example, reviews have found that apparent geographical variations could be explained by differences in study design [15]. Third, if challenge by diarrhoea pathogens can cause tropical enteropathy [16] without diarrhoea, a reduction in that challenge could reduce mortality risk without necessarily reducing diarrhoea morbidity.

In fact, the benefits to health of improving HSW are far greater than implied by disease-specific statistics. In the early 1900s, sanitary engineers in the US and Germany identified the “Mills-Reincke phenomenon.” Their studies showed that for every death from typhoid fever averted by water supply improvements, two to three deaths from other causes, including tuberculosis, pneumonia, and other causes of child mortality, were also avoided [17].

We now know that frequent bouts of diarrhoea and intestinal parasitosis are important causes of malnutrition, which renders children more susceptible to other diseases. For example, when malnourished children are recovering from an episode of diarrhoea, they are unusually susceptible to pneumonia; this diarrhoea-induced susceptibility may be associated with as much as 26% of all childhood pneumonia episodes [18]. Similarly, while 7% of the HSW-associated disease burden is directly associated with malnutrition, reductions in diarrhoea also reduce the incidence of diseases that are the consequence of malnutrition and that account for 29% of the disease burden (Figure 1).

**Figure 3:**
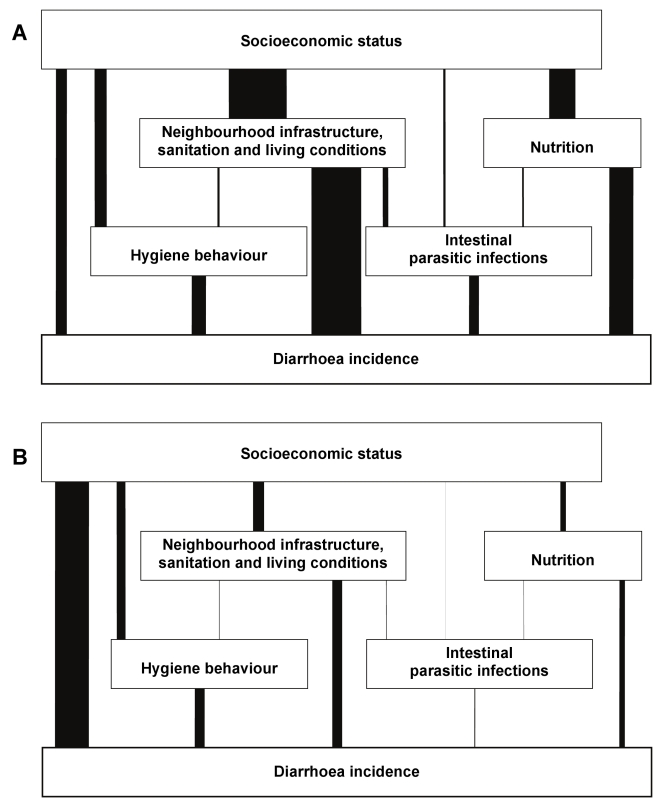
Determinants of diarrhoea in Salvador, Brazil, 1997–2004: Results of a hierarchical effect decomposition analysis. The width of each vertical bar shows the proportion of diarrhoea risk attributable to socioeconomic status and mediated by the intermediate variables shown. The two figures show conditions respectively (A) before and (B) after implementation of a major sanitation project. The project was associated with a 21% reduction in diarrhoea citywide, and 42% in the high incidence areas. Socioeconomic status accounted for 23% of the variance in diarrhoea rates before the project, but afterwards the strength of that link had been halved, to 11%. The proportion of that association mediated by intermediate variables, particularly sanitation, was also greatly diminished. Source: [20].

The disease burden weighs heavily on both households and health systems. It has been estimated that the health costs alone amount to some US$340 million for households lacking water supply and sanitation and US$7 billion for national health systems [19]. The household burden weighs most heavily upon the poor, but well-conceived sanitation and water programmes can weaken the link between poverty and disease [20] (Figure 3) and so contribute to health equity.

The World Bank/WHO Disease Control Priorities Project judged most interventions in HSW in developing countries to be highly cost-effective health interventions (Table 2). Indeed, hygiene promotion was the most cost-effective of all major disease control interventions at US$5 per DALY averted, with sanitation promotion also in the top ten at just over US$10 per DALY [21]. Although these figures do not consider the construction costs of water and sanitation facilities (which would lower cost-effectiveness if included) or the indirect costs of malnutrition (which would increase cost-effectiveness if included), Table 2 clearly shows that the HSW interventions most appropriate for the health sector are among the most cost-effective interventions it can make. Furthermore, most investments in water and sanitation infrastructure are made from other sources and for reasons other than health.

**Table 2:** Cost-effectiveness of HSW compared with other public health interventions.

Intervention	Cost-Effectiveness Ratio(DALYs Averted per US$1,000 Spent)
**Diarrhoeal disease**	
Hygiene promotion	200
Sanitation promotion	90
Water regulation and advocacy	12
Cholera or rotavirus immunization	0.5
**HIV/AIDS**	
Condom promotion and distribution	10–12
Antiretroviral therapy	1–3
**Malaria**	
Insecticide-treated bednets	80–140
Intermittent preventive treatment in pregnancy	120
**Tuberculosis**	
Directly observed short course (DOTS)	8–90

Source: [21].

## The Benefits of These Interventions Are Greater Than the Health Benefits Alone

**Figure 4:**
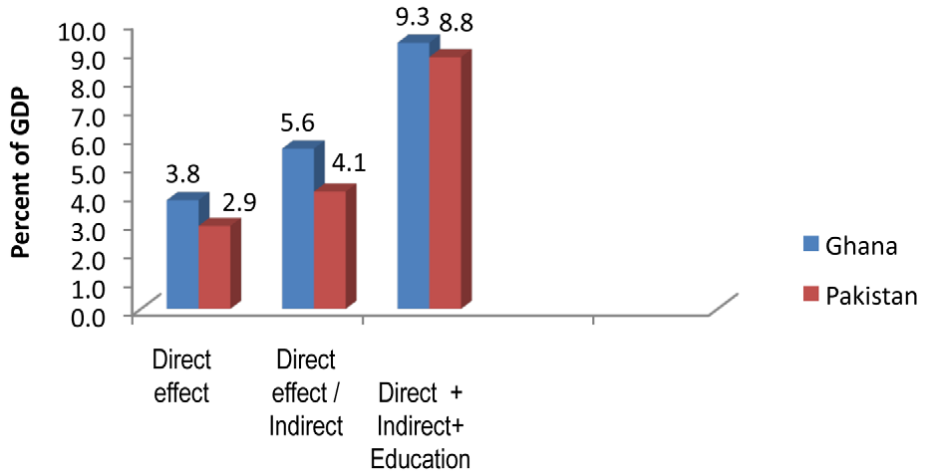
The cost to two national economies of inadequate HSW. The “direct” effect is mortality attributable to these environmental risk factors, “indirect” effect includes mortality mediated by environmentally caused malnutrition, and “education” includes the effects of that malnutrition on (i) grade attainment; (ii) school achievement (learning productivity) in terms of grade equivalents; (iii) delayed primary school enrolment; and (iv) grade repetition. The latter two effects result in delayed labour force entry. Source: [22].

Environmentally caused mortality and malnutrition have substantial economic costs. In Ghana and Pakistan, for example, the indirect effect on child mortality of environmental risk factors mediated by malnutrition adds more than 40% to the cost of directly caused child mortality (Figure 4) [22]. If one takes into account the effect of such malnutrition on impaired school performance and delayed entry into the labour market, the cost doubles to 9% of gross domestic product (GDP). With the possible exceptions of malaria and HIV/AIDS in Africa, it is hard to think of another health problem so prejudicial to household and national economic development.

Lack of sanitation also leads to intestinal helminth infections, which cause stunting, late entry to school, and impaired cognitive function [23],[24]. Furthermore, inadequate sanitation and water supply are associated with much loss of time spent on water collection or seeking a place to defecate. An analysis of survey data from 39 African countries showed that for 160 million people (many of them women), collection of each container of water took substantially more than 30 minutes [4],[25]. A World Bank study [26] found that, even ignoring the effect of water supplies on health, the value of time saved from water collection alone was sufficient to justify investments in rural water supply in most settings. Finally, a WHO report suggests that the time lost in collecting water and seeking somewhere to defecate could be valued at US$63 billion annually [27].

When all these benefits are accounted for, many HSW investments yield a net benefit in the range US$3–46 per dollar invested [19],[27], and some additional benefits remain unquantified. For example, there are suggestions that sanitation and water supply boost school attendance and reduce dropout rates—presumably in part by reducing the demand on children's time to collect water. Well-run sanitation facilities in schools might also help to prevent girls from dropping out after menarche [28]. Overcoming such constraints to education can yield real benefits. Thus, at the beginning of the 20^th^ century, 40% of schoolchildren in the southern US were infected with hookworm. When the disease was eradicated early in the century, school enrolment, attendance, and literacy increased, and there was a long-term gain in incomes [29].

These benefits are substantive at macroeconomic as well as household levels, as shown by the World Bank study cited above [22], and by a study for the Commission on Sustainable Development. This second study found that the per capita GDP growth of poor countries with improved access to water and sanitation was much higher than that of equally poor countries without improved access (3.7% and 0.1%, respectively) [30].

## The Ambition of the Millennium Development Goals Is Inadequate

In 2000, world Heads of State signed the Millennium Declaration, a global pact to reduce poverty. The associated Millennium Development Goals (MDGs) provide the policy framework and global benchmarks for this challenge.

The current international policy target for sanitation and water supply in MDG Target 7c aims to halve (between 1990 and 2015) “the proportion of the population without sustainable access to safe drinking water and basic sanitation.” The world is judged to be “on track” for drinking water access but “off track” for sanitation, for which it will miss the target by 1 billion people [4].

The attractive simplicity of the MDG target, which is based on categorising the world's households into “haves” and “have-nots” [31], contrasts with the diversity in levels of access and quality of service found on the ground (Table 3). While headline progress in increasing the proportion of households with sanitation is poor, the proportion of people defecating in the open is declining; and while progress on providing drinking water from improved public sources is on track, fewer people have a water supply at home than have basic sanitation at home, although sanitation is often referred to as “lagging behind water supply.” [4].

**Table 3:** Proportion of the population of developing countries with access at each level, in 1990 and 2008, to sanitation and water supply.

Level of Access	Proportion with Access (%)	
	1990	2008
**Excreta disposal**		
Open defecation	32	21
Unimproved	18	14
Shared	9	13
Improved	41	52
**Water supply**		
Unimproved	29	16
Other improved	32	35
House connection	39	49

Notes: “Unimproved” sanitation facilities are those with no hygienic separation of faeces from human contact; e.g. open pit, platform or bucket latrines. “Improved public” water sources include public taps or standpipes, tube wells or boreholes, protected dug wells and rainwater collection. “Piped water at home” means inside the user's dwelling, plot or yard. Source: [4].

Different levels of access provide widely varying health benefits. The change from open defecation to the use of an improvised latrine is a step forward, but is unlikely to offer health benefits unless the latrine provides an adequate barrier between the users and their excreta and is well maintained. Similarly, the health benefit of household water connections is substantially greater than that from an improved public source such as a protected well or standpipe (Figure 2).

Health benefits are also determined by the level and quality of service. For water supply, the MDG indicator is use of water from an improved source type, data for which is available from large-scale household surveys. However, households do not necessarily know about the quality of their water, so water safety is not accounted for. We do know that most water collected from improved public sources is contaminated with faeces by the time it is consumed [32] and that millions of people in Bangladesh use hand pumps on tube wells (i.e., “improved sources”) that provide water laced with arsenic [33].

The simplicity of headline indicators also masks wide geographic diversity. More than two-thirds of the population in Latin America, North Africa, and Southeast Asia, but only one-third in South Asia and sub-Saharan Africa, has improved sanitation. Globally, eight out of ten users of unimproved sanitation facilities, and six out of seven people who defecate in the open, live in rural areas. Moreover, low coverage does not always mean slow progress. South Asia has doubled the number of people with improved sanitation since 1990, and several African countries have increased the percentage served by more than 30% [4].

The MDG targets are themselves modest. They ignore the need for sanitation and water not only at home but also in schools, workplaces, and public places [31]. Even if the sanitation target is met, 1.6 billion people will still lack even a simple improved latrine at home. And if the drinking water target is reached in 2015, 800 million people will still live in homes where water is collected from distant or unprotected sources. The increase in the numbers of people with access is also being partly offset by population growth. Even if the target is met and the proportion of the unserved proportion is halved, neither the number of people unserved nor the global burden of disease will be halved.

The international community is likely to adopt further goals for HSW after 2015. In doing so, it will need to reconcile the compelling simplicity of a headline indicator (as in the present MDGs) with the need to encourage progressive improvement in levels and quality of service and comprehensive access at home, school, work, and public places. From the perspective of health, universal access to piped water and sanitation at home, school, and workplace must be the ultimate goal [31].

## HSW Continues to Have Health Implications in the Developed World

The impacts of poor HSW are not restricted to the developing world. Take the example of hand washing, which reveals an inappropriate level of complacency concerning hygiene in developed nations. Two intervention studies of hand washing with soap conducted in child-care centres in the US [34] and Australia [35] found reductions in diarrhoea of roughly 50%, similar to the reductions found in developing countries [11]. In another study, carers of young children in the UK washed their hands with soap after changing nappies on only 42% of occasions [36].

The idea that sanitation continues to have health implications in the developed world is a surprise to many. It should not be, given that flush toilets transport excreta but do not render it innocuous. Sewage treatment even in the most developed nations is not universal or fully effective, and effluent discharged into rivers and coastal areas constitutes a health risk to bathers, among others,. The costs of dealing with such effluent are considerable [37],[38].

The detection of disease outbreaks in developed nations also needs continued attention. In May 2000, a waterborne-disease outbreak in Walkerton, Canada (population 5,000) that involved more than 2,300 cases and at least seven deaths was traced to a small community water system. Researchers subsequently identified 99 disease outbreaks associated with public water supply systems and 138 in semipublic systems in Canada from 1974 to 2001. These findings drew attention to problems of data quality and the need for a national surveillance system for early detection of outbreaks [39]. Regrettably, detecting outbreaks does not prevent them. Small community systems are notoriously difficult to run properly but are far more common than often perceived. One in ten citizens of the European Union, for instance, receives their water from small or private systems [40]. However, waterborne disease outbreaks in the developed world are not confined to small systems. An outbreak in Milwaukee, US in 1993 that affected 400,000 people, caused more than 50 deaths, and cost an estimated US$96 million [41] was initially undetected by public health surveillance systems. Thus, surveillance does not always successfully prevent even massive outbreaks of waterborne disease.

## The Active Involvement of Health Professionals Is Crucial to Progress

The many non-health benefits mentioned above mean that the health sector is not alone in its interest in HSW. In practice, the main investments in HSW are made by ministries of water or education, by local governments and urban utilities, and by households themselves, which provide the opportunity for the health sector to influence large-scale deployment of resources for health.

It is tempting to hope that problems of HSW will quietly “go away” with economic development. It is tempting not least because it moves a health problem into someone else's backyard. Certainly, it is plausible to expect that economic development will be accompanied by satisfaction of demand for services such as water supply, and that this will lead to reductions in some water-related diseases. Unfortunately, evidence and experience show that these diseases are still with us, and suggest that health sector intervention is necessary to secure the fullest health benefits.

**Figure 5:**
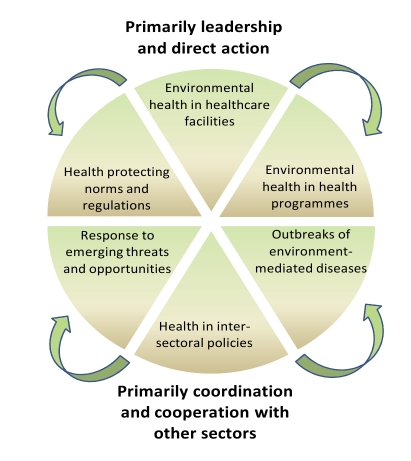
Health sector functions to secure environmental health. Source: [43].

There are specific functions that an effective health system must exercise to ensure effective environmental health including HSW (Figure 5) [42]–[44]. Some of these functions are led or acted on directly by health system entities, especially the provision of HSW in health care facilities, the investigation of outbreaks of HSW-related disease, and the integration of HSW into other health programmes. It is essential that those involved in disease-specific programmes incorporate HSW in their work. Thus, in one study, providing people living with HIV/AIDS with guidance on household water treatment and safe storage reduced the number of days they had diarrhoea by 33% [45]. Similarly, water systems in health care facilities can transmit opportunistic infections and legionellosis to high-risk groups. Simple control measures are effective, and health facility managers should be accountable for their consistent and effective implementation. There is much room for improvement. In a survey of 22 developing countries, 18%–64% of health care facilities were not disposing of waste properly [46].

Other functions require health professionals to engage intersectorally, particularly as advocates for health in intersectoral policy. Health professions must help to develop health-protecting norms and regulations, and must respond to emerging threats and opportunities. These are areas where the health system can have a strong influence on the delivery of safe services and technologies indirectly and at limited cost to the health sector itself. These functions present great opportunities to leverage resources for health. Unfortunately, all too often health professionals and institutions are insufficiently prepared for these functions.

The importance of the voice of health as an advocate for appropriate investments in HSW cannot be underestimated. In 19th century Britain, every major city had a Medical Officer of Health. These officials played a key role as advocates of clean and plentiful water supplies and sanitation as prerequisites for health. While it is hard to find quantitative evidence of their success, the qualitative case for their efficacy has been argued cogently [47].

To improve the current situation, five key tasks are required of health professionals: (1) maintenance and periodical replacement of existing services/facilities; (2) establishment of new services/facilities to cope with population growth; (3) provision of additional coverage to meet the MDG target and eventually achieve universal access; (4) progressive improvement of existing services/facilities to ensure that everyone benefits from the highest achievable standards; and (5) exposure of everyone, particularly the carers of young children, to well-conceived hygiene promotion.

All of these tasks require adequate financing, but only the last is normally directly implemented by health sector institutions. Health professionals therefore need to play a cross-sectoral role if they are to advocate effective investment.

Much more needs to be spent on HSW if these five tasks are to be completed. It has been estimated that annual investment by governments and aid agencies in water supply and sanitation in the developing countries totalled some US$15.7 billion in the 1990s, of which US$3.1 billion was for sanitation. The rate of investment has probably not increased much since then, but to carry out the second and third tasks alone would require an annual expenditure of around US$18 billion; the first task has been estimated to cost US$52 billion annually [48].

Currently, most expenditure on HSW is by individuals, through tariffs or building their own latrines. This dominance of household expenditure, which is a perceived norm in sanitation and water circles, contrasts with other aspects of preventive health care where State provision is advocated on the grounds of externalities. These are returns on investment which accrue to other people, or without the investor's knowledge. Households are typically more concerned with time-saving, privacy, convenience, and prevention of flooding than health, although health is in the interest of the community at large. Thus, interventions need to respond to the perceived needs of individuals and communities to ensure their sustainability.

## The Overlooked Foundation

Health evidence confirms that the burden of disease associated with inadequate HSW is overwhelmingly (although not exclusively) carried by the poor and disadvantaged in the developing world and is a major contributor to the cycle of poverty. Stated this way, HSW are *problems*.

Dealing effectively with HSW has the potential to reduce child mortality, one of the more recalcitrant health statistics, by a third. Investment in HSW in developing countries contributes to practically all of the MDGs, yields benefits that can be valued at many times their costs, and can reach even the poorest. Stated this way, HSW are *solutions*.

How well are national governments and donors responding to the challenge of providing HSW for all? Three statistics are especially telling. First, water and sanitation are the top priority for the poor. In participatory poverty assessments such as those carried out for national Poverty Reduction Strategic Plans (PRSPs), water appears among the top two priorities, even in apparently water-rich countries such as Papua New Guinea [49] and Uganda [50]. Second, despite the “Water for Life” decade, the International Year of Sanitation, and numerous regional interministerial conferences, sanitation is still accorded low priority. If sanitation appears at all in a national PRSP, it is usually with a zero budget allocation. Drinking water fares little better; in four out of five African countries studied, funds allocated in PRSP action plans (or related documents) did not match the importance of water issues noted in earlier descriptive parts of the same PRSPs [51]. Finally, despite commitments to target aid and to the “Paris Principles” of Aid Effectiveness [52], six of the ten countries in which more than half of the population live on less than a dollar a day receive less than the median aid per capita for sanitation and drinking water [53].

There is clearly much room for health professionals and health systems to do more for HSW, and an urgent need for them to do so. One of the really important things they can do is to engage more with other sector professionals with whom they share many goals such as the prevention of faecal-oral disease transmission. Moreover, health sector professionals are well placed to champion the massive changes in attitudes and practices required to progress HSW up the political ladder and out to everyone without good HSW services.

However, HSW implementation is not a single uniform process. Its three components are often implemented separately and by different agencies. Thus, the health promoters that encourage improved hygiene behaviours have little in common with the managers of piped water supplies, and the environmental health officers and sanitary technicians who support much basic sanitation need different skills from sewage treatment plant operators. What works and what does not are also very different across the three areas. For these reasons the next two papers in this series deal separately with water supply and with sanitation [54],[55] before the final paper reunites the threads to explore ways forward and lay out what needs to be done [56].

## Abbreviations

DALY: disability-adjusted life year
GDP: gross domestic product
HSW: hygiene, sanitation, and water supply
MDG: Millennium Development Goal
ORT: oral rehydration therapy
PRSP: Poverty Reduction Strategic Plan
